# Low-resolution pressure reactivity index and its derived optimal cerebral perfusion pressure in adult traumatic brain injury: a CENTER-TBI study

**DOI:** 10.1186/s13054-020-02974-8

**Published:** 2020-05-26

**Authors:** Lennart Riemann, Erta Beqiri, Peter Smielewski, Marek Czosnyka, Nino Stocchetti, Oliver Sakowitz, Klaus Zweckberger, Andreas Unterberg, Alexander Younsi, the CENTER-TBI High Resolution ICU (HR ICU) Sub-Study Participants and Investigators, Audny Anke, Ronny Beer, Bo-Michael Bellander, Andras Buki, Giorgio Chevallard, Arturo Chieregato, Giuseppe Citerio, Endre Czeiter, Bart Depreitere, George Eapen, Shirin Frisvold, Raimund Helbok, Stefan Jankowski, Daniel Kondziella, Lars-Owe Koskinen, Geert Meyfroidt, Kirsten Moeller, David Nelson, Anna Piippo-Karjalainen, Andreea Radoi, Arminas Ragauskas, Rahul Raj, Jonathan Rhodes, Saulius Rocka, Rolf Rossaint, Juan Sahuquillo, Ana Stevanovic, Nina Sundström, Riikka Takala, Tomas Tamosuitis, Olli Tenovuo, Peter Vajkoczy, Alessia Vargiolu, Rimantas Vilcinis, Stefan Wolf

**Affiliations:** 1 Department of Neurosurgery, Heidelberg University Hospital, INF 400, 69120 Heidelberg, Germany; 2 Brain Physics Laboratory, Division of Neurosurgery, Department of Clinical Neurosciences, University of Cambridge, Cambridge, UK; 3 Department of Physiopathology and Transplantation, Milan University, Milan, Italy; 4 Neuro ICU Fondazione IRCCS Cà Granda Ospedale Maggiore Policlinico, Milan, Italy; 5 Department of Neurosurgery, Klinikum Ludwigsburg, Ludwigsburg, Germany

**Keywords:** Cerebral autoregulation, Cerebrovascular reactivity, Cerebral perfusion pressure, CPPopt, Traumatic brain injury

## Abstract

**Background:**

After traumatic brain injury (TBI), brain tissue can be further damaged when cerebral autoregulation is impaired. Managing cerebral perfusion pressure (CPP) according to computed “optimal CPP” values based on cerebrovascular reactivity indices might contribute to preventing such secondary injuries. In this study, we examined the discriminative value of a low-resolution long pressure reactivity index (LPRx) and its derived “optimal CPP” in comparison to the well-established high-resolution pressure reactivity index (PRx).

**Methods:**

Using the Collaborative European NeuroTrauma Effectiveness Research in Traumatic Brain Injury (CENTER-TBI) study dataset, the association of LPRx (correlation between 1-min averages of intracranial pressure and arterial blood pressure over a moving time frame of 20 min) and PRx (correlation between 10-s averages of intracranial pressure and arterial blood pressure over a moving time frame of 5 min) to outcome was assessed and compared using univariate and multivariate regression analysis. “Optimal CPP” values were calculated using a multi-window algorithm that was based on either LPRx or PRx, and their discriminative ability was compared.

**Results:**

LPRx and PRx were both significant predictors of mortality in univariate and multivariate regression analysis, but PRx displayed a higher discriminative ability. Similarly, deviations of actual CPP from “optimal CPP” values calculated from each index were significantly associated with outcome in univariate and multivariate analysis. “Optimal CPP” based on PRx, however, trended towards more precise predictions.

**Conclusions:**

LPRx and its derived “optimal CPP” which are based on low-resolution data were significantly associated with outcome after TBI. However, they did not reach the discriminative ability of the high-resolution PRx and its derived “optimal CPP.” Nevertheless, LPRx might still be an interesting tool to assess cerebrovascular reactivity in centers without high-resolution signal monitoring.

**Trial registration:**

ClinicalTrials.gov Identifier: NCT02210221. First submitted July 29, 2014. First posted August 6, 2014.

## Background

Following severe traumatic brain injury (TBI), secondary injury cascades occur that affect cerebral blood flow (CBF). They may lead to ischemia when the cerebral perfusion pressure (CPP), the pressure gradient for cerebral blood flow defined as arterial blood pressure (ABP) minus intracranial pressure (ICP), is too low or to hyperemia and increased ICP when the CPP is too high [1–3]. The brain is vulnerable to changes in CPP after severe TBI because cerebral autoregulation, which normally maintains constant CBF during changes in ABP, is often impaired in those patients [1, 3–5]. A mainstay in the clinical management of TBI is therefore the avoidance of secondary brain injury by controlling ICP and ensuring adequate, non-harmful CBF by regulating CPP. Current guidelines (2016) by the Brain Trauma Foundation recommend keeping CPP between 60 and 70 mmHg [6]. However, likely due to the heterogeneity of cerebral injuries in patients with TBI, a CPP-oriented therapy with one fixed target for all patients failed to demonstrate improved neurological outcome compared to ICP-targeted therapy in a large randomized-controlled trial [7]. This is why a patient-customized approach has been proposed which uses the pressure reactivity index (PRx) to determine the optimal CPP (CPPopt) in an individual patient. The PRx, calculated as a moving correlation coefficient between slow waves of ABP and ICP, is a surrogate marker for cerebral autoregulation and has been associated with outcome after TBI in multiple studies [8–12]. Positive PRx values indicate dysfunctional cerebrovascular reactivity and are associated with increased mortality and unfavorable outcome, while negative values indicate intact pressure reactivity [9]. Using computational methods, this relationship can be exploited to determine an optimal CPP that corresponds to the lowest, most favorable PRx values in a patient [12, 13]. As the PRx and thus CPPopt are derived from ABP and ICP signals that are continuously monitored, the CPPopt recommendation can be constantly updated and refined, thereby providing the possibility to customize the clinical management also over the course of time in an individual patient. The automated CPPopt algorithm introduced by Aries et al. which uses a single, 4-h moving monitoring window to calculate CPPopt has been developed further to a multi-window algorithm. Deviations of CPP from CPPopt have been shown to correlate with clinical outcome in several retrospective studies [13–15], and the first prospective study assessing the feasibility of clinical management based on continuous determination of CPPopt is currently ongoing (COGiTATE trial) [16]. However, as the PRx and thus PRx-based CPPopt calculations require continuous, full-resolution waveforms of ABP and ICP, the CPPopt concept is currently limited to specialized neurocritical care units. In an attempt to increase accessibility of the PRx concept, a similarly calculated PRx variant called the long pressure reactivity index (LPRx) has been introduced which can be derived from lower resolution, minute-by-minute resampled ICP and ABP signals that standard monitoring devices in most intensive care units (ICU) can provide [17]. However, it remains unclear whether minute-by-minute monitoring might be of high enough resolution to evaluate autoregulation in patients with TBI and derive clinically relevant information from it. In fact, previous studies have yielded mixed results and drawn different conclusions [8, 17–19].

In light of these previous results, we assessed the discriminative value of LPRx and PRx and the performance of the most recent, multi-window CPPopt algorithm currently used in the COGiTATE trial but built on the LPRx instead of the PRx.

## Methods

### Patient cohort

For this study, all patients from the high-resolution (HR) ICU cohort of the Collaborative European NeuroTrauma Effectiveness Research in TBI (CENTER-TBI) study (EC grant 602150), prospectively recruited between January 2015 and December 2017 at 21 centers in the European Union (EU), were screened and included if they met the following inclusion criteria: (a) availability of high-frequency ICU monitoring data (i.e., continuous ABP and ICP monitoring), (b) availability of Glasgow Outcome Scale Extended (GOSE) at 6 months, (c) age of 18 years or older, and (d) ICP was measured via intraparenchymal probe. During the analysis, 8 patients were excluded from our study because PRx- or LPRx-based CPPopt could not be calculated due to either too short or interrupted monitoring data after artifact removal (3 patients) or very high mean ICP values over the entire monitoring period exceeding 55 mmHg and resulting in persistent PRx values close to + 1, making the CPPopt calculation meaningless (5 patients). For all remaining patients, the following demographic data was retrieved from the CENTER-TBI Neurobot database (version 2.0, CENTER core): age, sex, Glasgow Coma Scale (GCS), GCS–motor component, pupillary response at admission (bilaterally reactive, unilaterally reactive, bilaterally unreactive), and necessity of decompressive surgery (yes/no). For assessment of outcome, the imputed 6-month GOSE variable was used which includes both observed ratings and imputed values. For comparative analyses, patients were dichotomized into fatal vs. non-fatal outcome and unfavorable (GOSE 1–4) vs. favorable (GOSE 5–8) outcome.

### ICU monitoring data: recording and processing

High-frequency digital signals from continuous ABP and ICP monitoring during intensive care treatment were recorded for all patients included in this study. Recording was intended to start within 24 h of injury and encompassed the entire time that ICP/ABP monitoring was clinically required. ABP was monitored through radial or femoral arterial lines while ICP was monitored through intraparenchymal ICP probes, parenchymal fiber optic pressure sensors, or external ventricular drains. Signals were either digitally recorded or digitalized via an A/D converter (DT9801; Data Translation, USA MA), hereby sampled at frequency of 100 Hz or higher, using the ICM+ software (Cambridge Enterprise Ltd., UK), a Moberg CNS Monitor (Moberg Research Inc., USA), or a combination of both. Signal artifacts were removed manually and using automated algorithms. Data processing of all monitored signals was conducted using ICM+. Moving averages of 10 s (updated every 10 s) were calculated for ABP, ICP, and CPP (= ABP − ICP). PRx was calculated as a moving Pearson correlation coefficient between 10 s averaged ICP and ABP values in a moving time window of 5 min. For the LPRx, 1-min averages of ICP and ABP were correlated within a moving time window of 20 min. PRx, LPRx, and minute-by-minute averages of ABP and ICP were updated every minute. CPPopt was calculated using a multi-window, weighted approach. Details on the exact ICM+ settings and calculation methods can be found in the COGiTATE protocol paper by Beqiri et al. [16]. In short, a 5-min median CPP time trend was calculated along the PRx (Fisher transformed to achieve normal distribution) and these PRx values were grouped into CPP bins of 5 mmHg (within 40–120 mmHg range). The CPP value corresponding to the lowest associated PRx value was determined using an automatic parabolic curve fitting method. For each time point, this method was conducted for 36 different preceding time windows ranging from 2 to 8 h (with 10-min increasing steps) in length, resulting in 36 PRx-CPP plots. Following certain inclusion criteria (e.g., rejecting non-physiological values), those 36 different CPPopt values were combined according to particular heuristic weighting rules, which included higher weights given to more recent time windows and rejection of non-parabolic curves, to contribute to the final CPPopt. CPPopt_LPRx_ was calculated accordingly, however using the LPRx instead of PRx. CPPopt_PRx_ and CPPopt_LPRx_ were updated every minute. The difference between median CPP and each computed CPPopt_PRx_/CPPopt_LPRx_ value was continuously calculated for every minute of the recording period (ΔCPPoptL_PRx_, ΔCPPopt_LPRx_). The yield of CPPopt_PRx_/CPPopt_LPRx_ was defined and calculated as the count of CPPopt values divided by the count of CPP values across the whole recording period. Values of all monitoring parameters (ABP, ICP, PRx, LPRx, CPPopt_PRx_, CPPopt_LPRx_, ΔCPPopt_PRx_, ΔCPPopt_LPRx_) were at last averaged over the entire monitoring period for each patient.

### Statistical analysis

Demographical variables and ICU monitoring parameters were descriptively analyzed and compared between dichotomized outcome groups using the Mann-Whitney *U* test for continuous variables and the chi-squared test for categorical variables. Results are given as median + interquartile range (IQR), unless stated otherwise. Spearman’s rank correlation coefficient was used to evaluate correlation between PRx and LPRx while a Bland-Altman plot was used to assess agreement between both indices. Univariate logistic regression in regard to dichotomized outcomes was applied with either LPRx or PRx as predictors. Discrimination was assessed via area under the receiver operating curve (AUC) and compared using DeLong’s test. Multivariate logistic regression was conducted to assess the association between either LPRx or PRx and dichotomized outcomes with adjustment for the IMPACT core variables age, admission GCS motor score (GCS-Motor), and pupil reactivity status (bilaterally reactive, unilateral reactive, bilateral unreactive) [20]. The goodness-of-fit of the full model (IMPACT core variables + LPRx or PRx) was tested against the IMPACT-only model via a likelihood-ratio test, and AUCs were calculated for each model. In all statistical tests, the level of significance was set at 0.05, and no *p* value adjustment for multiple testing was applied due to the exploratory nature of this study. All statistical analyses were performed in the R environment [21].

## Results

### Patient demographics

A total of 224 patients (176 males and 48 females) ranging in age from 18 to 85 years with a median age of 51 (IQR, 33–64 years) were included in this study. Patient demographics and mean values of ICU monitoring parameters are provided in Table 1. The median GCS score at admission was 6 (IQR, 3–10) with a range from 3 to 15. Decompressive craniectomy was performed in 47 patients during their hospital stay. Six months after admission, 52 patients were dead yielding a mortality rate of 23%. The outcome of 135 patients (60%) was considered unfavorable (i.e., GOSE 1–4) at 6 months post-injury.

**Table 1:** Patient demographics

	All patients
**Number of patients**	224
**Age (years)**	51 (33–64)
**Sex**	
**- Female (%)**	48 (21%)
**- Male (%)**	176 (79%)
**GCS total**	6 (3–10)
**GCS motor**	4 (1–5)
**Pupillary response**	
**- Both reactive**	150 (67%)
**- One reactive**	17 (8%)
**- Both unreactive**	39 (17%)
**- Unknown**	18 (8%)
**ABP (mmHg)**	83.6 (78.8–90.0)
**ICP (mmHg)**	12.6 (9.6–16.1)
**CPP (mmHg)**	71.6 (65.2–77.4)
**PRx**	0.027 (− 0.074–0.162)
**LPRx**	− 0.049 (− 0.145–0.079)
**GOSE**	3 (3–5)
**Unfavorable outcome (%)**	135 (60%)
**Fatal outcome (%)**	51 (23%)

*ABP* arterial blood pressure, *CPP* cerebral perfusion pressure, *GCS* Glasgow Coma Scale, *GOSE* Glasgow Outcome Scale Extended, *ICP* intracranial pressure, *LPRx* long pressure reactivity index, *PRx* pressure reactivity index

### PRx and LPRx

**Fig. 1:**
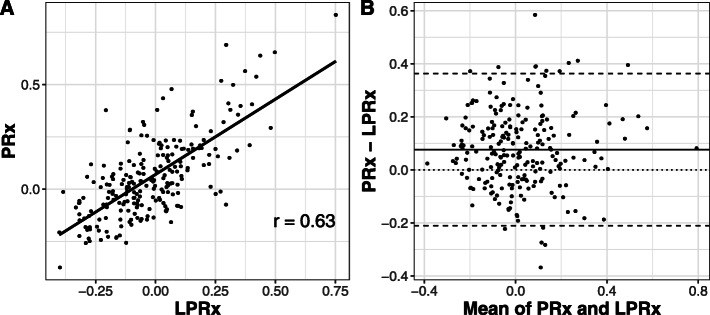
**a** Spearman’s correlation between LPRx and PRx. **b** Bland-Altman plot for agreement between PRx and LPRx showing mean bias (solid line), zero difference (thin dashed line), and 95% lines of agreement (thick dashed lines)

**Fig. 2:**
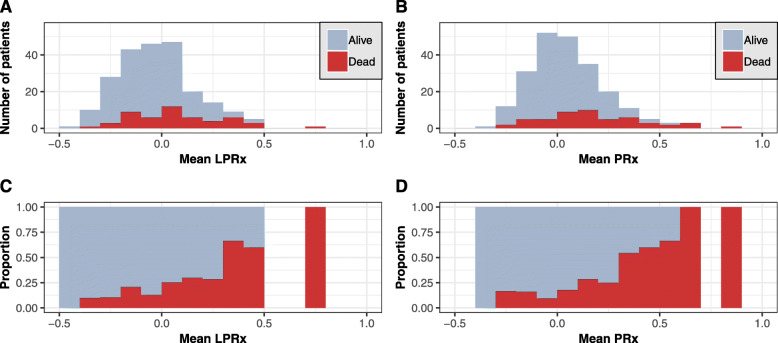
Histograms showing the absolute (**a**, **b**) and relative (**c**, **d**) proportion of patients with fatal outcome for different LPRx and PRx values

**Fig. 3:**
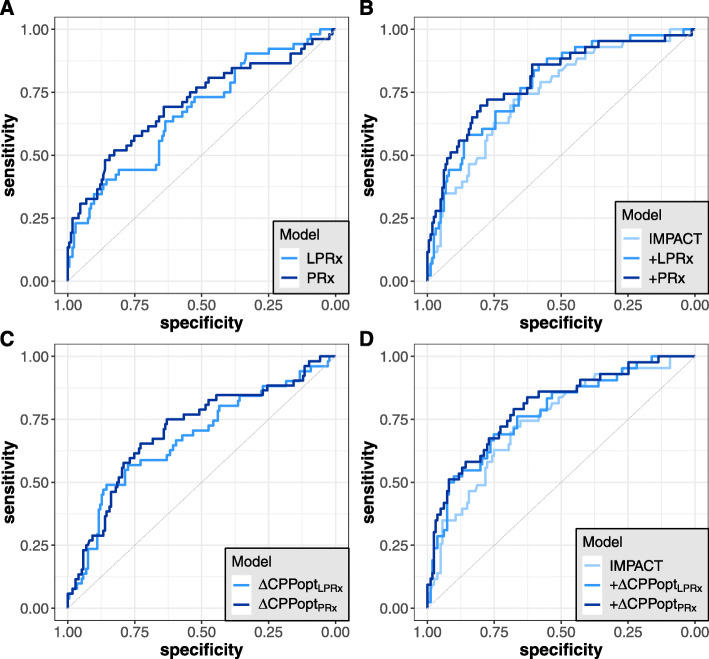
AUCs for the prediction of mortality for different regression models. **a** LPRx and PRx. **b** IMPACT variables with the addition of LPRx and PRx. **c** ΔCPPopt_LPRx_ and ΔCPPopt_PRx_. **d** IMPACT variables with the addition of ΔCPPopt_LPRx_ and ΔCPPopt_PRx_

Results from Spearman’s test showed that there was a significantly positive, albeit only moderate correlation between mean LPRx and PRx values in our patient cohort (*r* = 0.63, *p* < 0.001; Fig. 1a). This correlation was slightly higher for the subgroup of patients with severe TBI (*r* = 0.68, *p* < 0.001). A Bland-Altman plot for agreement between LPRx and PRx yielded a mean bias of 0.076 with 95% limits of agreement at − 0.210 and + 0.362 (Fig. 1b). There was a clear association of higher mortality rates with higher mean LPRx and PRx values, as shown in Fig. 2. Accordingly, both LPRx and PRx were significantly higher in patients with fatal outcome compared to surviving patients (LPRx, 0.025 (− 0.096–0.212) vs. − 0.064 (− 0.175–0.054), *p* < 0.001; PRx, 0.163 (0.016–0.314) vs. 0.009 (− 0.082–0.112), *p* < 0.001). When dichotomizing patients into unfavorable vs. favorable outcome, a significant group difference could only be detected for the PRx but not for the LPRx (PRx, 0.052 (− 0.067–0.195) vs. − 0.014 (− 0.086–0.098), *p* = 0.013; LPRx, − 0.026 (− 0.142–0.105) vs. − 0.063 (− 0.148–0.051), *p* = 0.204). In univariate regression analysis, both PRx and LPRx were significantly associated with mortality (Table 2 (A)), which generally increased with higher index values. Discriminative ability was higher for the PRx (AUC 0.70 (0.61–0.79), *p* < 0.001) compared to the LPRx (AUC 0.66 (0.58–0.75), *p* < 0.001), although this difference did not reach significance in this patient cohort (DeLong’s test, *p* = 0.348; Fig. 3a). Interestingly, discriminative ability of both reactivity indices seemed to be particularly strong for the subgroup of patients with severe TBI (LPRx: AUC 0.69 (0.57–0.81) and PRx: AUC 0.73 (0.62–0.84)). The discriminative value in regard to unfavorable outcome was considerably worse for both indices, and only the PRx reached significance in univariate regression with an AUC of 0.60 (0.52–0.67, *p* = 0.007). However, in comparison to the AUC of LPRx (0.55 (0.47–0.63, *p* = 0.102)), no significant difference could be observed via DeLong’s test either (*p* = 0.167).

**Table 2:** Results from univariate and multivariate logistic regression analysis in regard to mortality for LPRx, PRx, ΔLPRx-CPPopt, and ΔPRx-CPPopt

A	Parameter	**Index: LPRx**		**Index: PRx**	
		Coefficient	*p* value	Coefficient	*p* value
Univariate	*Index*	3.484	**< 0.001**	4.244	**< 0.001**
Multivariate	*Age*	0.039	**0.001**	0.036	**0.003**
	*GCS-Motor*	− 0.191	0.068	− 0.202	0.059
	*Pupillary response*	0.304	0.197	0.333	0.173
	*Index*	2.634	**0.011**	3.868	**< 0.001**
B	Parameter	Δ**CPPopt: LPRx**		Δ**CPPopt: PRx**	
		Coefficient	*p* value	Coefficient	*p* value
Univariate	Δ*CPPopt*	− 0.122	**0.003**	− 0.148	**< 0.001**
Multivariate	*Age*	0.049	**< 0.001**	0.050	**< 0.001**
	*GCS-Motor*	− 0.196	0.059	− 0.227	**0.034**
	*Pupillary response*	0.308	0.198	0.257	0.296
	Δ*CPPopt*	− 0.139	**0.003**	− 0.159	**< 0.001**
C	Parameter	**Index: LPRx**		**Index: PRx**	
		Coefficient	*p* value	Coefficient	*p* value
Univariate	*Index*	3.484	**< 0.001**	4.244	**< 0.001**
Multivariate	*Age*	0.043	**< 0.001**	0.039	**0.002**
	*GCS-Motor*	− 0.164	0.130	− 0.169	0.127
	*Pupillary response*	0.457	0.067	0.474	0.068
	*ICP*	0.071	**0.005**	0.061	**0.019**
	*CPP*	0.034	0.128	0.035	0.128
	*Index*	1.628	0.152	3.140	**0.007**
D	Parameter	Δ**CPPopt: LPRx**		Δ**CPPopt: PRx**	
		Coefficient	*p* value	Coefficient	*p* value
Univariate	Δ*CPPopt*	− 0.122	**0.003**	− 0.148	**< 0.001**
Multivariate	*Age*	0.050	**< 0.001**	0.051	**< 0.001**
	*GCS-Motor*	− 0.166	0.124	− 0.190	0.086
	*Pupillary response*	0.466	0.067	0.423	0.106
	*ICP*	0.067	**0.009**	0.065	**0.011**
	*CPP*	0.042	0.066	0.046	**0.048**
	*Index*	− 0.108	0.065	− 0.146	**0.002**

*ΔCPPopt* difference between cerebral perfusion pressure and calculated optimal cerebral perfusion pressure, *GCS-Motor* Glasgow Coma Scale motor component, *LPRx* long pressure reactivity index, *PRx* pressure reactivity index

**Fig. 4:**
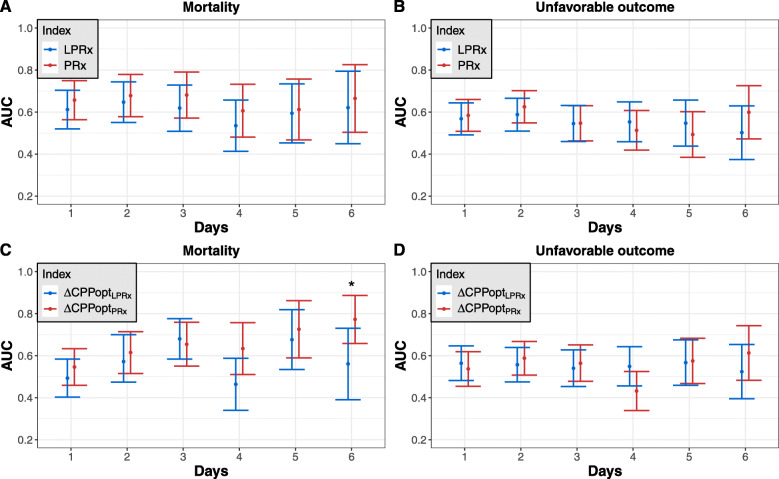
AUCs for the prediction of mortality and unfavorable outcome over the early post-traumatic time course. **a** LPRx and PRx in regard to mortality. **b** LPRx and PRx in regard to unfavorable outcome. **c** ΔCPPopt_LPRx_ and ΔCPPopt_PRx_ in regard to mortality. **d** ΔCPPopt_LPRx_ and ΔCPPopt_PRx_ in regard to unfavorable outcome. The only significant difference was observed at day 6 between LPRx and PRx (*p = 0.007)

Similar results were obtained when examining the predictive value of the reactivity indices in relation to patient outcome over the post-traumatic temporal course (Fig. 4a, b). Discrimination tended to be higher for PRx compared to LPRx for both mortality and, with exceptions at days 4 and 5, unfavorable outcome. However, those differences did not reach significance at any observed time point.

When adjusted for age, GCS-Motor, and pupillary response (IMPACT core variables) in multivariate regression analysis, both LPRx and PRx remained independent predictors for mortality (Table 2 (A)). In regard to unfavorable outcome, neither of the indices was found to be a significant predictor in the same multivariate model (PRx: *p* = 0.120 and LPRx: *p* = 0.976). Importantly, when also controlling for ICP and CPP in the multivariate model, only PRx but not LPRx remained significant (Table 2 (C)). Nevertheless, adding either LPRx or PRx to the IMPACT core model led to a notable increase of the AUC for prediction of mortality: 0.78 (0.70–0.85) when adding LPRx and 0.80 (0.72–0.88) when adding PRx, compared to 0.74 (0.66–0.82) for the model without indices (Fig. 3b). For both LPRx and PRx, the goodness-of-fit of the combined IMPACT + index model was significantly improved when compared to the mere IMPACT model as assessed by likelihood-ratio tests (*χ*^2^ = 6.79, *p* = 0.009 (LPRx), and *χ*^2^ = 15.13, *p* < 0.001 (PRx), respectively). Results and trends in our analysis were similar when patients with decompressive surgery were excluded (data not shown).

### Optimal cerebral perfusion pressure

Both the LPRx-based and the PRx-based CPPopt calculations produced a similar median CPPopt value in our patient cohort (71.4 (65.9–76.6) mmHg vs. 72.0 (65.9–77.5) mmHg, *p* = 0.445). Correspondingly, we found no significant difference in CPPopt yield between the LPRx- and PRx-based calculations (80.0 (70.4-86.7) % vs. 80.4 (71.4–87.6) %, *p* = 0.625). The difference between CPP and CPPopt_PRx_ (ΔCPPopt_PRx_) was significantly higher in patients with fatal outcome compared to survivors (3.7 (1.9–5.8) mmHg vs. 1.9 (0.9–4.1) mmHg, *p* = 0.003). This relation was also apparent when examining the ΔCPPopt_LPRx_, although it was considerably lower (2.3 (1.0–3.6) mmHg vs. 1.5 (0.7–2.9), *p* = 0.049). ΔCPPopt_LPRx_ and ΔCPPopt_PRx_ were significant predictors of mortality both in univariate and multivariate analyses with age, GCS-Motor, and pupillary as covariates, underlining the association between both CPPopt versions and clinical outcome (Table 2 (B)). However, when also including ICP and CPP in the multivariate model, only ΔCPPopt_PRx_ remained a significant predictor (Table 2 (D)). Still, there was no significant difference between the AUCs of ΔCPPopt_LPRx_ and ΔCPPopt_PRx_ when predicting mortality (0.67 (0.59–0.76) ΔCPPopt_LPRx_ vs. 0.71 (0.62–0.79) ΔCPPopt_PRx_; *p* = 0.237; Fig. 3c). Assessment of the discriminative value of both ΔCPPopt variables over the post-traumatic course did reveal neither a clear increase/decrease over time nor a clear superiority of one ΔCPPopt variable over the other. However, a significant statistical difference could be observed at day 6, with ΔCPPopt_PRx_ displaying a significant higher AUC compared to ΔCPPoptL_PRx_ when predicting death (*p* = 0.007, DeLong’s test; Fig. 4c, d). Similar to our findings with the LPRx and PRx, the goodness-of-fit was significantly improved when adding either ΔCPPopt_LPRx_ or ΔCPPopt_PRx_ to the basic IMPACT model (*χ*^2^ = 9.39, *p* = 0.002 (IMPACT + ΔCPPopt_LPRx_), and *χ*^2^ = 16.71, *p* < 0.001 (IMPACT + ΔCPPopt_PRx_); Fig. 3d). The LPRx plus IMPACT model displayed an AUC of 0.77 (0.69–0.85), and the PRx plus IMPACT model an AUC of 0.80 (0.72–0.87). Similar to our previous findings with the PRx and LPRx, equivalent results were obtained for all CPPopt-associated analyses without including surgically decompressed patients, except for results from the multivariate models including ICP and CPP where ΔCPPopt_LPRx_ remained a significant predictor when only including patients without decompressive craniectomy (data not shown).

### Cerebral perfusion pressure targeted therapy

**Fig. 5:**
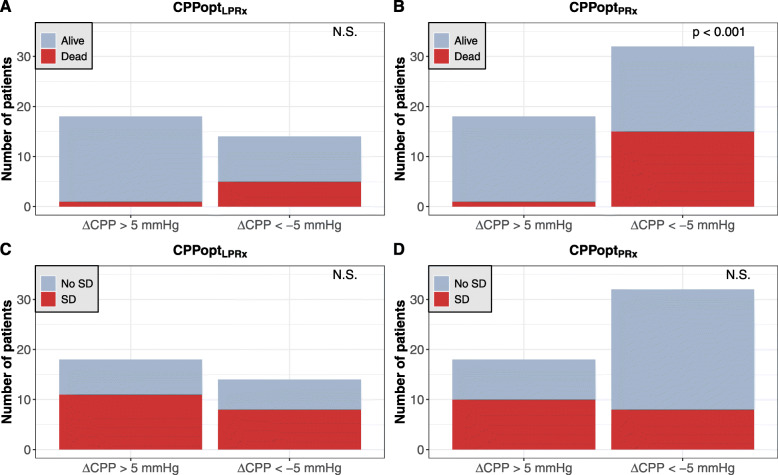
Bar graphs showing the proportion of fatal outcome (**a**, **b**) and severe disability (**c**, **d**) in patients with a mean deviation of at least 5 mmHg below or above their CPPopt_LPRx_ or CPPopt_PRx_

Because a difference of ± 5 mmHg between the actual CPP and calculated CPPopt has been proposed as an acceptable range for CPP-targeted therapy [12, 16], we analyzed outcome in a subgroup of patients whose CPP deviated on average by at least ± 5 mmHg from their calculated CPPopt_LPRx_ (*n* = 32) and CPPopt_PRx_ (*n* = 50, Fig. 5a**–**d). Of 18 patients with a mean ΔCPPopt_PRx_ > 5 mmHg (“hyperperfused” patients), only one patient died (6%). In contrast, 15 of 32 patients with a mean ΔCPPopt_PRx_ < − 5 mmHg (“hypoperfused” patients) were dead at the 6-month follow-up (47%). The mortality rates differed significantly between those groups (*χ*^2^ = 7.24, *p* = 0.007). According to the calculated CPPopt_LPRx_, a mean ΔCPPopt_LPRx_ > 5 mmHg was present in 18 patients with the same mortality rate of 6%. However, only 14 patients were determined to be on average at least 5 mmHg below their CPPopt_LPRx_ (ΔCPPopt_LPRx_ < − 5 mmHg), and of those, 5 had died (35%). The mortality rates when comparing “hypo- and hyperperfused” patients according to CPPopt_LPRx_ missed statistical significance (*χ*^2^ = 2.93, *p* = 0.087).

Eight of 32 patients (25%) with a mean ΔCPPopt_PRx_ < − 5 mmHg were severely disabled after 6 months. This proportion was distinctively higher in patients with a mean ΔCPPopt_PRx_ > 5 mmHg with 10 of 18 patients (56%), although the rates of severe disability between those two patient groups did not reach statistical significance (*χ*^2^ = 3.44, *p* = 0.064). Similar results were found for the CPPopt_LPRx_: 3 of 14 patients (27%) with a mean ΔCPPopt_LPRx_ < − 5 mmHg and 10 of 18 patients (56%) with a mean ΔCPPopt_LPRx_ > 5 mmHg were severely disabled 6 months after injury (*χ*^2^ = 2.52, *p* = 0.113). Again, similar results were obtained in all statistical tests when only patients without decompressive surgery were considered (data not shown).

## Discussion

To individualize therapy is a promising concept for possible reduction of mortality and unfavorable outcome in patients with TBI. Regulating CPP according to computed CPPopt recommendations derived from cerebrovascular reactivity indices might hereby play an important role. However, it remains unclear if high-resolution data for PRx calculation is necessary to obtain relevant CPPopt values or if low-resolution, minute-by-minute signals for LPRx calculation might suffice. This question is particularly important because using the LPRx would make the CPPopt concept available to a wider range of centers and thus patients. The first two pilot studies examining the LPRx showed encouraging results, stating that LPRx seemed to perform equally well in outcome prediction and CPPopt calculation as the PRx [17, 18]. However, the validity of those studies was limited by relatively small patient numbers (18 and 29, respectively). More recently, a larger (over 300 patients) single-center follow-up study concluded the low-resolution LPRx to be less precise compared to PRx in outcome prediction which our results seem to confirm [8]. The proposed weaker discriminative ability could be further supported by our data especially when ICP and CPP were also added to the IMPACT variables in a multivariate model, where PRx but not LPRx remained a significant predictor. The reason for this inferior performance is likely that the LPRx only includes slower drifts in ABP and ICP which provide less information on the state of autoregulation, as opposed to the PRx which also includes higher frequency wave components, likely representing more outcome-relevant autoregulatory responses.

However, even when performing to some extent worse than the PRx in outcome prediction, the LPRx almost always showed significant results in our analyses as well and the differences of AUCs between PRx and LPRx in univariate regression to mortality were non-significant in our current work (albeit in a smaller sample). This was also true when comparing the discriminative value of both indices day-by-day during the early post-injury time course (first 6 days).

What is very important is that both indices significantly improved the performance of a multivariable model containing the IMPACT core variables and remained independent predictors for mortality after TBI. However, LPRx lost significance when also adjusting for ICP and CPP. Nevertheless, when taken together, those results seem to support the notion proposed by previous studies [19, 22] that minute-by-minute averaged signals, while performing to some extent weaker, might still carry important outcome-related information and might be sufficient for autoregulation monitoring via pressure reactivity indices. Notably, both indices performed considerably worse when predicting unfavorable outcome compared to predicting mortality, which is also in accordance with previous studies [10, 11]. A subgroup analysis showed an especially strong association with outcome for LPRx/PRx in patients with severe TBI, making our results particularly applicable for such patients.

Given the abovementioned findings, we sought to evaluate the performance of a weighted, multi-window algorithm for assessing CPPopt that is based on the LPRx instead of the PRx. Determination of CPPopt has the potential to translate the PRx/LPRx concept into clinical management by offering dynamic management targets for CPP according to CPPopt. Ideally, patients might then directly benefit from this individualized therapy. The CPPopt concept built on PRx values has been shown to be of prognostic value in numerous studies in the sense that deviations of CPP from CPPopt were predictors of fatal outcome in TBI patients [12–15, 19, 23]. While the first automated CPPopt algorithm was based on PRx/CPP values in a moving single-window of 4 h for calculation [13], this method was extended to include multiple windows for calculations by Depreitere et al. [19], who used minute-by-minute monitoring data and various low-resolution indices for their approach. They could show that the resulting CPPopt was highly related to outcome and was not inferior in outcome prediction compared to the single-window CPPopt_PRx_ which is based on high-resolution data.

The multi-window concept developed by Depreitere et al. was then adapted to high-resolution data, and thus PRx, and extended with additional weighting and safety criteria as well as more calculation windows in an algorithm implemented in ICM+ by Liu et al. [14], and subsequently modified further to make it suitable for clinical, bedside application as part of the COGiTATE trial [16]. In our study, we sought to evaluate how the low-resolution LPRx, instead of the PRx, would perform in this most recent CPPopt calculation method. Using the CENTER-TBI high-resolution ICU cohort, we were able to do this in direct reference to the PRx-based approach and in a multi-center dataset. Similar to the LPRx itself, CPPopt_LPRx_ performed slightly worse in outcome prediction than its PRx counterpart but was still a significant predictor for mortality in univariate and multivariate analysis including the IMPACT variables. Both ΔCPPopt_LPRx_ and ΔCPPopt_PRx_ were significant predictors of mortality, even when patients were adjusted for other prognostic factors in multivariate analysis, and their addition to the IMPACT core model could significantly improve the goodness-of-fit. Addition of ΔCPPopt displayed even higher AUCs for mortality than addition of reactivity indices to the model. Interestingly, when also including ICP and CPP in addition to the IMPACT variables in the model, ΔCPPopt_PRx_ remained significant while ΔCPPopt_LPRx_ failed to demonstrate significance in the entire cohort. However, ΔCPPopt_LPRx_ remained significant in non-decompressed patients, indicating a potential use in this patient group. All those results seem to emphasize the importance that the deviation of CPP from CPPopt might have in regard to clinical outcome. Further studies should examine the extension of the IMPACT core model with autoregulation monitoring indices, analogous to the already present IMPACT core + CT model or IMPACT core + CT + laboratory markers model.

When only patients with a relevant mean CPP/CPPopt deviation were considered, the CPPopt_PRx_ method detected more patients overall and showed a closer relation to mortality in patients with an average deviation in CPP of at least 5 mmHg below the PRx-CPPopt. Even the CPPopt_LPRx_ showed a substantially higher mortality rate in “hypoperfused” patients when compared to “hyperperfused” ones, although this difference between mortality rates did not reach significance. Similar to Aries et al. [13], we found a higher rate of severe disability in “hyperperfused” patients compared to “hypoperfused” ones. However, this association did not reach significance in our study.

Concerning our results on CPPopt availability (yield of the algorithm), it has to be mentioned that CPPopt_PRx_/CPPopt_LPRx_ could not be calculated in 8 patients, for reason not related to a failure of the CPPopt algorithm. In 5 patients, ICP was so high and CPP so low that the autoregulation was completely and entirely lost, and the concept of “optimal” CPP was therefore not applicable. In further 3 patients, there was simply not enough data available to perform the calculation. In all the remaining patients, the fraction of time where CPPopt could be determined was importantly very similar between CPPopt_LPRx_ and CPPopt_PRx_.

Interestingly, the exclusion of patients who underwent decompressive surgery during their ICU stay did not affect the results in most of our analyses (data not shown), similar to previously published results in other studies. A notable exception is that ΔCPPopt_LPRx_ remained a significant predictor for mortality in a multivariate model including ICP and CPP only in non-decompressed patients while miss significance in the entire cohort. This is despite the fact that the performance of PRx and thus also LPRx, as the pressure reactivity monitor, depends on reliable transmission of changes in cerebral blood volume into intracranial pressure [24, 25], and that is theoretically adversely affected by the decompressive craniectomy. However, the exact timing of decompressive surgery could play an important role as indices are averaged over the whole monitoring period and future studies should be conducted to examine this relationship closer.

### Limitations

As the CENTER-TBI study was designed to be a prospective observational study, treatment strategies and protocols in ICUs might considerably differ between participating centers and might therefore be confounders. Importantly, ICP and mean arterial pressure (MAP) signals were subject to manipulation by treating clinicians (e.g., actively lowering ICP or MAP in selected patients) and might therefore be also the result of therapeutic interventions. Moreover, this study contains a heterogenous group of patients in terms of demographics, injury characteristics, and comorbidities which might influence the results. Finally, the considered variables such as the indices and differences between CPP and CPPopt were averaged over the entire monitoring time per patient which could mean that their variability and the presence of short periods with very deviated values were not accounted for in our analysis. Regarding the temporal course of the discriminative power of both indices, it is important to note that the sample size considerably decreased over time which might influence the results especially at later time points. While this multi-center study can provide evidence for the relevance of LPRx and CPPopt_LPRx_, a high-quality, prospective study is needed to conclude whether the CPPopt_LPRx_ concept can be translated into clinical benefit in patients with TBI.

## Conclusions

Our findings indicate that the LPRx and multi-window CPPopt_LPRx_ do not reach the precision of the PRx and CPPopt_PRx_ in outcome prediction after TBI. However, the LPRx is still significantly associated with outcome and can produce outcome-relevant CPPopt values, most reliably in non-decompressed patients. A prospective trial is needed to assess if this association is strong enough for a meaningful clinical translation. Should the ongoing COGiTATE feasibility/safety trial and subsequent phase 3 trials be successful in proving the CPPopt concept, it might be worthy to consider a respective trial with the CPPopt_LPRx_ to make the CPPopt concept available to a broader range of centers and patients.

## Data Availability

Data from the CENTER-TBI study is available to researchers after submitting a detailed study proposal (https://www.center-tbi.eu/data.) that is approved by the CENTER-TBI management committee. Data from this study is available from the corresponding author upon reasonable request and with permission of the CENTER-TBI management committee.

## Abbreviations

ABP: Arterial blood pressure
AUC: Area under the curve
CBF: Cerebral blood flow
CPP: Cerebral perfusion pressure
CPPopt: Optimal cerebral perfusion pressure
ICP: Intracranial pressure
ICU: Intensive care unit
IQR: Interquartile range
GCS: Glasgow Coma Scale
GOSE: Glasgow Outcome Scale Extended
MAP: Mean arterial pressure
LPRx: Long pressure reactivity index
PRx: Pressure reactivity index
TBI: Traumatic brain injury
